# Causes and consequences of coagulation activation in sepsis: an evolutionary medicine perspective

**DOI:** 10.1186/s12916-015-0327-2

**Published:** 2015-05-06

**Authors:** Maiara Marx Luz Fiusa, Marco Antonio Carvalho-Filho, Joyce M Annichino-Bizzacchi, Erich V De Paula

**Affiliations:** Faculty of Medical Sciences, University of Campinas, Rua Tessália Vieira de Camargo 126, Cidade Universitária Zeferino Vaz, 13083-878 Campinas, SP Brazil; Hematology and Hemotherapy Center, University of Campinas, Campinas, SP Brazil

## Abstract

**Background:**

Coagulation and innate immunity have been linked together for at least 450 million years of evolution. Sepsis, one of the world’s leading causes of death, is probably the condition in which this evolutionary link is more evident. However, the biological and the clinical relevance of this association have only recently gained the attention of the scientific community.

**Discussion:**

During sepsis, the host response to a pathogen is invariably associated with coagulation activation. For several years, coagulation activation has been solely regarded as a mechanism of tissue damage, a concept that led to several clinical trials of anticoagulant agents for sepsis. More recently, this paradigm has been challenged by the failure of these clinical trials, and by a growing bulk of evidence supporting the concept that coagulation activation is beneficial for pathogen clearance. In this article we discuss recent basic and clinical data that point to a more balanced view of the detrimental and beneficial consequences of coagulation activation in sepsis.

**Summary:**

Reappraisal of the association between coagulation and immune activation from an evolutionary medicine perspective offers a unique opportunity to gain new insights about the pathogenesis of sepsis, paving the way to more successful approaches in both basic and clinical research in this field.

## Background

Sepsis has been recently defined as a “life threatening condition that arises when a body’s response to an infection injures its own tissues and organs” [1]. The last decades have witnessed continuous improvements in our understanding of the pathogenesis of this condition. Nonetheless, sepsis is still associated with mortality rates as high as 30% [2], and with a 10% annual increase in its incidence [2], it remains one of the biggest challenges of modern medicine [3].

The host response to an invading pathogen is one of the key determinants of patient outcome in sepsis [4]. This concept has been acknowledged for decades, based on the limitation of antibiotic therapy for sepsis treatment, and is supported by increasing knowledge about the cellular and molecular pathways involved in host:pathogen interaction [4,5]. Virtually all living beings have developed sensing mechanisms for rapid detection of invading pathogens, which in humans are represented by pattern-recognition receptors (PRR). These receptors recognize conserved motifs in pathogens as well as danger signals that indicate cellular stress in sterile inflammatory conditions [6,7]. As expected, receptors and ligands of this sensing system have been under strong selective pressure during primate and hominid history [8,9], with pathogens regarded as key determinants of how these molecules evolved. An illustration of this concept is provided by the demonstration of convergent evolution of Toll-like receptor (TLR) genes in European and Rroma (Gypsy) populations sharing the same geographic region. Similarities in TLR genes were found between these two distinct populations which were exposed to plague in Europe during the Middle Ages, when compared to people from northwest India, which is the geographic origin of the Rroma population [10]. Because sepsis severity is mediated by the host response to a pathogen, it is not surprising that variations in these and other immune-related genes can influence sepsis severity [11].

However, the cellular processes activated by invading pathogens during sepsis are not restricted to classical immune-related genes, but also involve pathways less intuitively related to the immune system. Pathways that regulate endothelial barrier integrity and hemostasis (coagulation activation) are two important examples. The former facilitates access of leukocytes to tissues through diapedesis, contributing to pathogen clearance. In regard to the latter, discussing the proximate reasons of why coagulation is activated in sepsis, and the consequences thereof, are the two main objectives of this review. The importance of these two questions lies in the fact that the intensity and regulation of coagulation activation in sepsis seems to play a major role in the determination of patient outcome: be it pathogen clearance (and cure), or the development of secondary tissue damage such as disseminated intravascular coagulation (DIC) or multiple organ dysfunction [12].

In order to address these important questions from an evolutionary perspective, we will first discuss some of the potential selective pressures that molded the hemostatic system of modern humans. Next, we will confront the classical view about the role of coagulation activation during sepsis with emerging experimental data that could help us answer the important “why” question about the evolutionary reasons for coagulation activation during sepsis. Finally, we will discuss how the views that emerge from this discussion could influence research on sepsis.

## Discussion

### Selective pressures molding the hemostatic system

While pathogens are recognized as the main selective pressure on immune-related genes such as TLR and others [9,13], much less is known about the pressures that influenced the evolution of the hemostatic system. In humans, the ultimate goal of hemostasis is to keep blood from leaving the intravascular space through acquired breaches in endothelial lining. This is achieved by a finely regulated system capable of rapidly responding to the contact of tissue factor (TF), a protein that is normally segregated to the extravascular space, with coagulation factors present in plasma. This system is organized in a “cascade” fashion involving platelets, leukocytes, and pro- and anticoagulant proteins, that act in concert to promote the formation of clots that seal breaches in the vascular endothelium until tissue is repaired.

Biochemical and molecular evidence support the idea that the current organization of the human hemostatic system evolved more than 450 million years ago [14]. Although little information on the selective pressures that guided this evolution is available, one can speculate that increasing complexity of body plan organization may have been an important factor. Hemostatic systems in invertebrates such as the chelicerate horseshoe crab or in *Drosophila* are composed of much fewer genes and proteins than in vertebrates, but they present similarities that indicate a common origin of at least part of their components. For example, clot stability depends on the action of a transglutaminase that appears to contribute to clotting in all invertebrates, and is homologous to human factor XIIIa [15]. In animals with more complex body plan organization, constraints on the diffusion of oxygen and other vital molecules, as well as compartmentalization of body functions in different organs, required the development of closed circulatory systems, with organ-specific adaptations of vascular bed anatomy and physiology. This complexity is based on significant heterogeneity of endothelial cell phenotype throughout the vascular tree [16,17] and is also associated with variations in hemostatic function between arteries and veins and even within venous and arterial beds. Organ-specific variations of hemostasis are well illustrated by clinical differences in the expression of thromboembolic diseases in different organs [18].

In addition to adaptations to increasingly complex circulatory systems, the close association between hemostasis and the innate immune response is another factor that influenced the evolution of the human hemostatic system. Evidence that hemostasis and inflammation evolved from a single-triggered mechanism can be traced back more than 450 million years, based on studies with the horseshoe crab (*Limulus polyphemus*) [19]. Trauma to the exoskeleton is a major threat to these invertebrates that possess a rapid cell-based cascade-like response system, able to form an extracellular clot in the event of any breach to their integument. Since these ancient eukaryotes live around ocean waters, evolution selected a system triggered by minimum concentrations of bacterial endotoxin, present in Cyanobacteria (blue-green algae) for more than 2 billion years [20]. Interestingly, the extreme sensitivity of this system is the basis of the laboratory assay used to detect endotoxin contamination, which relies on proteins originated from these invertebrates. Horseshoe crabs do not possess a circulatory system, and the same endotoxin-triggered system protects these animals from both infection and loss of the internal milieu after injury. In humans, more complex and independent systems exist for the protection against pathogen invasion and bleeding. Nevertheless, the sharing of common initiators (such as endotoxin), as well as the overlap between several pathways, indicate the close association between the evolution of hemostasis and of the immune system during the last 450 million years.

Infectious diseases such as smallpox, bubonic plague, and malaria are recognized as important selective pressures acting upon the immune system genes in the last 10,000 to 20,000 years [8,21]. However, different selective pressures must have been relevant to innate immunity and hemostasis evolution in the remaining 2.5 million years of *Homo* evolution [22]. During this long period, much lower population densities and the nomadic habits of hunter-gatherers did not allow the same patterns of pathogen spread observed in post-agricultural societies, so that trauma, and not contagious infectious disease, was the most important cause of death [21]. In line with these observations, a comprehensive necropsy program performed in a national park in Tanzania identified trauma, rather than infection, as the main cause of death among chimpanzees in the wild [23]. Similarly, predation-associated injuries were identified as a key selective pressure among anthropoid primates [24]. In an environment in which trauma caused by conflict or outdoor activities had to be dealt with without the aid of modern healthcare, a quick-responding and highly effective hemostatic system was most likely under a strong positive selection pressure. Moreover, in the absence of basic hygiene knowledge and antibiotics, it is easy to picture the importance of hemostasis as part of the innate immune system, whereby fibrin, platelet and leukocyte-rich clots contribute to avoid pathogen spread, as stated in the “hemostatic containment” hypothesis [25].

In this context, any trait that optimized hemostasis and wound repair without increasing the risk of thrombosis must have been beneficial in evolutionary terms. Given the very low prevalence of modern risk factors for thromboembolic diseases in ancient times, the trade-off equation between bleeding and thrombosis may have favored the development of an extraordinarily effective hemostatic system for ancient challenges, but one that, when exposed to modern stimuli such as high-fat diet, smoking, sedentarism, and ageing, contributes to the high prevalence of thromboembolic diseases [26]. The emergence and fixation of factor V Leiden in northern Europe about 30,000 years ago is a good illustration of this concept. Factor V Leiden is a genetic polymorphism that increases an individual’s hemostatic capacity, resulting in less bleeding during delivery. However, when combined with the exogenous estrogen therapy present in oral contraceptives, it increases the incidence of venous thromboembolism [27]. The fact that these selective pressures on the hemostatic system operated for much longer periods than recent epidemics of plague and smallpox, maybe even before the divergence of humans from other great apes, may explain why modern tools used to detect selection signatures in our genome tend to identify the immune system, and not hemostasis, as a main target of natural selection. Accordingly, variations favoring a highly efficient hemostatic system may have been fixed in our genomes for millions of years, and may be less conspicuous to these statistical tools. Interestingly, *KNG1*, the gene encoding kininogen, which is regarded as one of the main contacts between hemostasis and inflammation, was recently shown as a target of long-lasting selective pressure [28].

### Classical view of the role of coagulation activation in sepsis

The recent market withdrawal of recombinant activated protein C (rhaPC), a natural anticoagulant used in the treatment of sepsis, represents the last chapter of a story characterized by serial failures of large-scale clinical trials designed to test the generally accepted assumption that coagulation activation and microvascular thrombosis were major determinants of tissue damage in sepsis [29]. The earliest evidences about the role of coagulation activation during sepsis included histological demonstration of microvascular thrombosis in target organs of septic patients and the progressive decrease in platelet counts and coagulation factor levels in the late stages of sepsis, attributed to a “consumption coagulopathy”. In the following years, the bulk of the experimental data indicated that sepsis was indeed associated with a shift in the hemostatic balance towards a procoagulant state. The most convincing data were: (i) the demonstration that tissue factor expression in circulating leukocytes can be stimulated by pathogens; (ii) an acquired deficiency of endogenous anticoagulant proteins such as antithrombin and protein C in sepsis patients; and (iii) a sustained increase in fibrinolysis inhibitors such as PAI-1, resulting in hypofibrinolysis [30]. Together, these data supported the concept that coagulation activation was at least in part responsible for the organ failure observed in sepsis. Manipulation of coagulation in animal models of sepsis provided further support to this concept, by showing that organ failure and even mortality could be limited by the blockade of discrete elements of hemostasis such as tissue factor [31] and factor VII [32], among others.

Based on this model, ambitious clinical development programs of recombinant natural anticoagulants (antithrombin, TFPI, and rhaPC) in patients with sepsis were launched, going all the way to phase 3 trials and, in one case, market approval. Unfortunately, the benefits of this strategy could not be confirmed in these trials, although additional clinical trials and meta-analysis are warranted before a definite conclusion on this issue can be reached [33]. Limitations of animal models [34] and in clinical trial design [29] have both been cited as potential explanations for the dissociation between preclinical and clinical data. We could also contribute to this discussion, by arguing for a more cautionary view of the long-standing assumption of a direct cause-and-effect relationship between coagulation activation and multi-organ failure in sepsis. In fact, the presence of microvascular thrombosis in target organs in sepsis was only demonstrated in studies with a limited number of patients, which did not consider the different phases of sepsis in their analysis [35,36]. More recent autopsy studies confirming these findings are restricted to very small case series of patients with fulminant sepsis [37,38]. In addition, although we do not argue against the presence of some degree of systemic hypercoagulability in sepsis, a concept supported by the recent demonstration of sepsis as an independent predictor of venous and arterial thrombosis [39,40], the presence of this phenomenon in the early stages of sepsis has been challenged by data generated using global hemostasis tests, which pointed to a consistent down-regulation of thrombin generation in the early stages of sepsis [41,42]. In this context, a reappraisal of old and new data using an evolutionary medicine framework [43] can refine our understanding about the ultimate and proximate causes of hemostasis activation during sepsis.

### Why is coagulation activated during sepsis?

The concept that coagulation activation can be beneficial during infections was suggested several years ago [20] and is gaining increasing support from high-quality data generated during the last decade. Excellent reviews about studies linking coagulation and innate immunity have been recently published [4,44,45]. Our goal here is to present an updated summary of these studies, highlighting their interpretation from an evolutionary medicine perspective.

Several studies suggest that coagulation proteins are necessary for eradication of invading pathogens. It is now known that tissue factor also triggers coagulation-independent signaling pathways mediated by protease activated receptors (PARs) on immune cells [46]. These PAR-dependent signals evoke pro- and anti-inflammatory pathways that regulate migration and proliferation of immune cells, angiogenesis, endothelial adhesion, and several other components of the host response to an infection [47,48]. PAR-dependent pathways are also activated by other components of hemostasis such as activated protein C, factor Xa, and thrombin [46], increasing the list of coagulation factors that regulate immune function.

The contact system, formerly known as the initiator of the intrinsic pathway of coagulation, is also involved in the host response to pathogens. Kininogen, one of the elements of the contact system, is now recognized as an important source of antimicrobial peptides released upon the recognition of several microorganisms by this protein [49]. Of note, a study looking for genomic signatures of positive selection has recently shown that kininogen has been under strong selective pressure during evolution [28]. Other components of hemostasis, such as coagulation factors II, X, and fibrinogen, have also been shown to release antimicrobial peptides, not necessarily involved with blood clotting (reviewed in [44]).

Another mechanism by which coagulation activation contributes to pathogen clearance is by forming a physical barrier that circumscribes infection foci, thereby facilitating pathogen clearance by immune cells. This “containment hypothesis” [25] is now supported by several lines of evidence indicating that down-regulation of different components of hemostasis (such as fibrin and platelets) hampers pathogen clearance (Table 1). A convincing demonstration that fibrin can be protective during infections comes from a study with fibrinogen-deficient mice, which presented increased mortality and enhanced bacterial growth in a model of *Listeria monocytogenes* infection. The role of fibrin formation in the protective effect was confirmed by the reproduction of these results in mice treated with warfarin, an anticoagulant that down-regulates fibrin formation by an alternative mechanism [50]. A similar strategy confirmed the role of thrombin generation and fibrin formation in *Yersinia enterocolitica* infection [51]. Impaired pathogen clearance in fibrinogen-deficient mice was also suggested in a study using a group A streptococcal infection model [52]. Factor XIII, regarded as the most evolutionary conserved coagulation factor [53], also seems to be important for pathogen clearance, as suggested by an elegant study showing that *Streptococcus pyogenes* are immobilized and killed inside fibrin clots, in a factor XIII-dependent fashion [15].

**Table 1:** **Effect of coagulation factor deficiencies in animal models of sepsis**

**Model (genetic alteration)**	Effect on hemostasis*	**Sepsis/infection model**	**Effect on pathogen clearance/sepsis severity**
**EPCR deficiency**	↑	Pneumococcal sepsis	↓ bacterial dissemination [81]
		Melioidosis**	Unaltered [82]
		Endotoxemia	↑ mortality [83]
**α2-antiplasmin deficiency**	↓	Melioidosis**	↑ mortality, ↑ bacterial dissemination [59]
**Overexpression of EPCR**	↓	Pneumococcal sepsis	↑ bacterial dissemination [81]
		Melioidosis**	↑ bacterial dissemination [82]
**t-PA deficiency**	↑	Melioidosis**	↓ mortality, ↓ bacterial dissemination [56]
		Septic peritonitis	↑ mortality, ↑ bacterial dissemination [84]
**Factor XI deficiency**	↓	*Y. enterocolitica* (ip)	Unaltered [51]
		Peritoneal sepsis	↓ mortality [71]
		Listeriosis	↓ mortality, ↓ bacterial dissemination [70]
**Factor VIII deficiency**	↓	Endotoxemia	Unaltered [85]
		*E. coli* (ip)	↑ bacterial growth, ↔ survival [86]
**Factor IX deficiency**	↓	Endotoxemia	Unaltered [85]
**PAI-1 deficiency**	↓	Melioidosis**	↑ mortality, ↑ bacterial dissemination [57]
		H. influenza infection	↑ bacterial dissemination [58]
		Klebsiella pneumonia	↑ mortality, ↑ bacterial dissemination [87]
**Fibrinogen deficiency**	↓	*Y. enterocolitica* (ip)	↑ mortality, ↑ bacterial dissemination [51]
		Group A streptococci	↑ mortality [52]
		Listeriosis	↑ mortality, ↑ bacterial dissemination [50]
**Factor V deficiency**	↓	Group A streptococci	↑ mortality [52]
**Tissue factor deficiency**	↓	*S. aureus* sepsis	Unaltered [72]
		*Y. enterocolitica* (ip)	↑ mortality [51]
		Endotoxemia	↓ inflammation [88]
		Endotoxemia	↓ mortality, ↓ inflammation [31]
**PAI-1 + TAFI double deficiency**	↓	*Y. enterocolitica* (ip)	↑ mortality [51]
**Factor II deficiency**	↓	*S. aureus* sepsis	↓ mortality [72]
**Factor XIII deficiency**	↓	*S. aureus* sepsis	Unaltered [72]
		*S. pyogenes* (skin)	↑ bacterial dissemination [15]
**Protein C deficiency** (het)	↑	Endotoxemia	↑ mortality [89]
**TAFI deficiency**	↓	*E. coli* (ip)	Transient ↑ bacterial outgrowth [90]
**Factor VII deficiency**	↓	Endotoxemia	↓ mortality, ↓ inflammation [32]
**Factor V leiden**	↑	Septic peritonitis	Unaltered [91]
		Endotoxemia	↓ mortality [76]***

*The global effect of each genetic deficiency on hemostasis was defined based on current knowledge about hemostatic mechanisms. **Respiratory sepsis by intranasal instillation of Gram-negative *Burkholderia pseudomallei*. ***Lower mortality observed only in heterozygous mice. EPCR: endothelial protein C receptor. t-PA: tissue-type plasminogen activator; PAI-1: plasminogen activator inhibitor-1; TAFI: thrombin-activatable fibrinolysis inhibitor; ip: intraperitoneal; het: heterozygous.

The fibrinolytic system, which regulates hemostasis function by the degradation of fibrin thrombi when they are no longer necessary, also seems to be involved in pathogen clearance. It has been known for several years that sepsis is associated with impaired fibrinolysis, attributed to a brisk rise of a fibrinolysis inhibitor (PAI-1) in plasma [54,55]. While for several years this hypofibrinolytic state was viewed as one of the causes of microvascular thrombosis and tissue damage, an alternative explanation would be that by shutting down fibrinolysis, the host could limit the spread of invading pathogens by more resistant fibrin clots. Studies with genetically modified mice support the latter explanation. Accordingly, mice with tissue-type plasminogen activator deficiency, which results in impaired fibrinolysis, presented lower bacterial growth in the primary site of infection (lungs) in a murine model of Gram-negative sepsis [56]. In line with this observation, deficiencies of fibrinolysis inhibitors PAI-1 [57,58] and α2-antiplasmin [59], both of which are associated with increased fibrinolytic activity, resulted in impairment of pathogen clearance in models of bacterial and viral infections. Another very significant evolutionary clue comes from the several examples of pathogen virulence factors based on proteases that degrade fibrin clots [60], of which streptokinase from *Streptococcus pyogenes* [61] and a plasminogen activator from *Yersinia pestis* are only the most famous examples [62,63].

Low platelet counts have long been recognized as an important prognostic factor in sepsis, based on the assumption that they were a biomarker of sepsis severity. In this regard, we recently demonstrated that platelet turnover, measured by the immature platelet fraction, correlates with sepsis severity [64]. However, recent studies indicate that platelets are in fact important players in host defense. Wong et al. demonstrated that platelets interact with Kupffer cells to encase blood-borne pathogens in the liver [65]. In another interesting study, antibody-induced thrombocytopenia resulted in impaired survival and in a proportional increase in bacterial growth in a model of pneumonia-derived sepsis [66]. The now widely recognized participation of neutrophil extracellular traps (NETs) in host defense [67,68] and the role of platelet activation in NET formation [69] provide another link between hemostasis and innate immunity [45].

In spite of all these evidences, one should bear in mind the complexity of the interactions between hemostasis, innate immunity, and pathogens. For yet unknown reasons, factor XI deficiency in mice, which is also associated with reduced fibrin formation, has been consistently shown to improve, rather than hamper, host response in different models of infection [70,71]. Survival advantage observed in studies with coagulation factor-deficient mice are not always consistently observed when different models of infection are used [72]. And in some settings, the so-called arms race between pathogen and host seems to have turned fibrin as an asset, rather than a limitation for some pathogens [72]. Indeed, this complexity is well illustrated by the heterogenous effect of the down-regulation of thrombin generation/clot strength in sepsis outcome in different animal models (Table 1).

Clinical data also contributed to the idea that some level of coagulation activation could be beneficial to pathogen clearance, in that initial studies suggesting that coagulation inhibition could limit tissue damage during sepsis were not confirmed in phase 3 clinical trials of anticoagulant agents for these patients [73-75]. The impact of factor V Leiden (FVL), a procoagulant variant of coagulation factor V, in sepsis mortality is also worth discussing. When analyzed in the population of a large phase 3 sepsis study, carriers of FVL presented asignificantly lower 28-day mortality than non-carriers [76]. Although this data could not be confirmed by other studies [27], the observation at least challenges the concept of a detrimental effect of coagulation activation during sepsis.

While the laboratory and clinical evidence presented so far point to a beneficial role of coagulation activation during sepsis, new evidence supporting the classical paradigm that coagulation activation can contribute to tissue damage in sepsis has also been published. *In vivo* microvascular imaging studies demonstrated disturbances of tissue perfusion in patients with sepsis, which could be reverted by the use of the anticoagulant activated protein C [77,78]. In addition, the negative results of randomized clinical trials of anticoagulant agents in sepsis have been challenged by recent systematic reviews and clinical trials [79,80], suggesting a beneficial effect of this treatment strategy in subgroups of patients with sepsis. If confirmed (in ongoing clinical trials and in larger meta-analysis [33]), these results point to the existence of a threshold above which coagulation activation becomes detrimental during sepsis.

## Summary

### Ultimate causes of coagulation activation in sepsis and implications for future research

**Figure 1:**
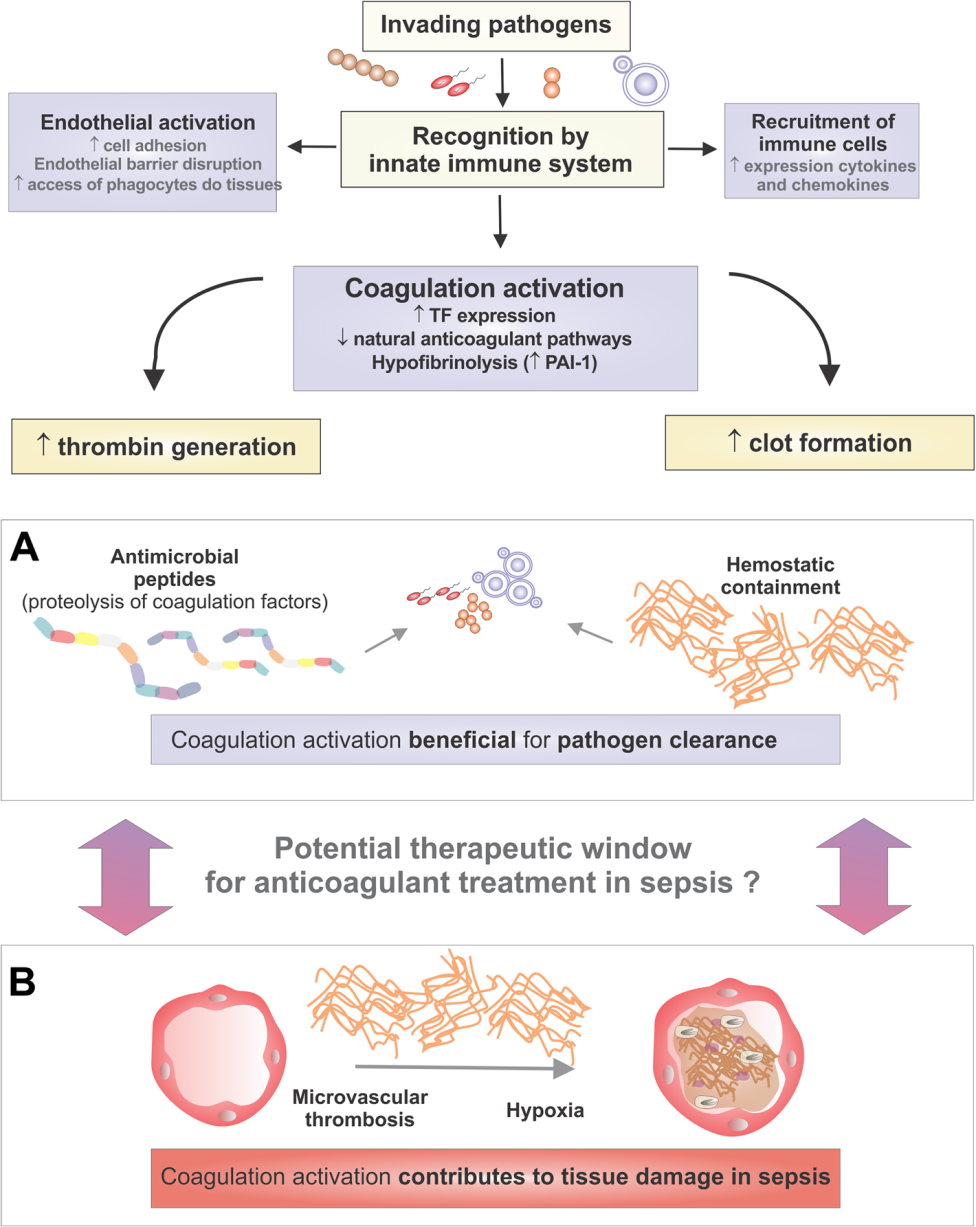
**Potential consequences of coagulation activation during sepsis.** Recognition of invading pathogens by innate immunity triggers mechanisms that contribute to pathogen clearance such as endothelial activation and recruitment of immune cells to infection sites. Coagulation activation is part of this stereotyped response. During sepsis, increased tissue factor expression, down-regulation of natural anticoagulant pathways, and hypofibrinolysis result in increased thrombin generation and clot formation. The beneficial consequences of coagulation activation for pathogen clearance are depicted in panel **A** and include the release of antimicrobial peptides from the proteolysis of several proteins of the coagulation cascade, and limitation of pathogen spread by fibrin-mediated hemostatic containment. However, deregulated coagulation activation could also contribute to microvascular thrombosis and hypoxia, thereby contributing to tissue damage in sepsis. The precise identification of the moment when coagulation activation turns from a beneficial to a detrimental process would allow more rational therapeutic approaches for sepsis, preserving the ancient link between hemostasis and innate immune response. PAI-1: plasminogen activator inhibitor-1.

Together, these data suggest that coagulation activation is an important component of the overall response against invading pathogens, and that eradication of invading pathogens could be considered the ultimate cause of coagulation activation during infection and sepsis. From this point of view, one can understand why several individual compartments of hemostasis are tuned towards the generation of increased amounts of thrombin during sepsis, based on the importance of fibrin and platelets in the host response to infection. The analysis of coagulation activation during sepsis from this evolutionary medicine perspective could also contribute to the explanation of why the use of systemic anticoagulants were not beneficial in large-scale trials in sepsis, highlighting the importance of identifying the precise moment at which coagulation activation turns from a beneficial to a detrimental process in sepsis (Figure 1). In this context, any targeted treatment for sepsis that limits coagulation activation should be tailored to preserve the function of a host defense mechanism that seems to have been around for at least 450 million years.
